# Water-based air purifier with ventilation fan system: a novel approach for cleaning indoor/outdoor transitional air during the pandemic

**DOI:** 10.1007/s42452-022-05142-5

**Published:** 2022-09-05

**Authors:** Arnon Jumlongkul

**Affiliations:** School of Medicine, Mae Fah Luang University, Chiang Rai, 57100 Thailand

**Keywords:** COVID-19, Haze, Particulate matter, Transition air, Water-based air purifier

## Abstract

**Abstract:**

This article presents the design and fabrication of an air purifier that uses a water-based technique to clean indoor/outdoor transitional air to provide a low-tech air purifier against the annual smog crisis and the ongoing COVID-19 pandemic. The air purifier was designed and built. All tests were conducted in a closed room as well as a semi-outdoor area. Particle sizes of PM0.3, 0.5, 1.0, 3.0, 5.0, and 10 μm (particle/m^3^) were measured at an air inlet, air outlet, 2 m from an air inlet, and 4 m from an air outlet after 0, 5, 10, 15, and 20 min of air treatment, respectively, as well as CO_2_ levels and relative humidity (RH). The average airflow rate was also measured. When compare to 0 min, all parameters, except semi-outdoor PM0.3 and CO_2_ levels, tend to decrease in both indoor and semi-outdoor conditions. When measure by total airflow specification of a dual ventilation fan, the average airflow rate at an air outlet is reduced by 20 times.

****Article Highlights**:**

- Design and fabrication of a water-based air purifier.
- A low-tech air purifier helping to protect against the annual smog crisis and the ongoing COVID-19 pandemic.
- The novel water-based air purifier effectively traps air particles ranging in size from 0.5 to 10 µm.

## Introduction

Particulate matter (PM) is a mixture of solid and liquid particles suspended in air. Its size, chemical, physical, and biological properties change depending on where it is and when it is measured. PM can have an immediate and long-term impact on health problems [1]. Acute PM2.5 exposure can trigger an inflammatory response, oxidative stress, and apoptosis in lung tissue [2]. Long-term exposure to PM2.5 may increase the incidence of cardiovascular diseases, renal disease, type 2 diabetes mellitus, and chronic obstructive pulmonary disease (COPD) [3]. PM also increases the risk of myocardial infarction, stroke, cardiac failure, coronary heart disease, pneumonia, acute respiratory distress syndrome, renal failure, hepatic injury, cerebrovascular diseases, gastrointestinal disorders, and inflammation in humans [4]. Air pollution can harm the nervous system by increasing oxidative stress, activating microglia cells, and causing brain damage [5]. Increased PM10 and PM2.5 levels in children have been linked to an increased risk of outpatient department (OPD) visit for respiratory illnesses [6]. There was a link between PM2.5 exposure and depression, anxiety, and suicide as part of the mental health effects [7]. Furthermore, exposure to PM is associated with an increase in hospital admissions for mental disorders as well as the economic burden of hospitalization for mental disorders [8].

According to [9], the minimum size of a COVID-19 containing respiratory particle is about 4.7 µm, while SARS-CoV-2 genes can be found in aerosols at 0.25–0.5 µm. Despite the fact that Coronaviruses are quite small, measuring 65–125 nm in diameter [10]. SARS-CoV-2 aerosol particles, which are typically less than 10 µm in size, can remain suspended in air for hours and can be transported up to several meters from of the source, a particular issue in asymptomatic people [11]. As part of saliva droplets, a study model revealed that the initial size of droplet vectors, measuring 20–50 µm, resulted in 4.7–12 µm solid residues within a few seconds [12]. Furthermore, scientists discovered some links between the SARS-CoV-2 virus and PM. For indoor conditions, ventilation systems are the primary means of controlling virus spread, whereas outdoor risk sources, such as aerosolized particles from wastewater treatment and PM, can become virus carriers [13]. In terms of biology, SARS-CoV-2 has been shown to have a high affinity for the angiotensin-converting enzyme 2 (ACE2) receptor, whereas PM exposure increases ACE2 expression in the lungs. As a result, in heavily polluted areas, PM may facilitate pulmonary SARS-CoV-2 viral adhesion [14]. Therefore, air cleaning processes can limit the spread of the COVID-19 virus as well as PM.

Since we know this virus may spread through the air, scientists have attempted to develop multiple technologies to protect humans from it. Numerous techniques have been used to improve indoor air quality (IAQ), such as an indoor plantation to promote a green environment, purified air circulation, maintaining social distance, wearing face masks, portable air purifiers with filters (e.g. activated carbon, HEPA filters, ionization, photo-catalytic oxidation (PCO), germicidal UV radiation), humidification, adequate ventilation, and lockdown policies [15]. Because of inefficient conventional air filters, Heating, Ventilation, and Air–conditioning (HVAC) systems, which are installed as part of air conditioners, may aid in the spread of infectious indoor air. An innovative air circulation concept should combine HEPA or ULPA filters, the use of Ultraviolet Germicidal Irradiation (UVGI) and/or ionization in the airstream, and a moisture control system (relative humidity (RH) in the 40–60% range is recommended) [16]. A systematic review found that the risk of outdoor SARS-CoV-2 transmission was low (< 10%), and the probability of indoor transmission was very high when compared to outdoor transmission [17]. As a result, air purifiers for open spaces may be ineffective. However, transitional areas between indoor and outdoor air, also known as “semi-outdoor” as well as large-open places that are always crowded with people (e.g. platforms, front doors of large buildings, halls, large canteens), have been highlighted. In this area, critical information about the effectiveness of air treatment has been scarcely available. High-performance filtration technologies may be inappropriate under these conditions.

As stated previously, the goal of this article is to focus on the design and fabrication of an air purifier, using a water-based technique for cleaning indoor/outdoor transitional air, as well as to test its efficiency, in order to provide a low-tech water-based air purifier, both against the smog crisis and the COVID-19 pandemic. The following section of the article discusses how to build the water-based air purifier as well as testing methods. Section 3 presents the results of laboratory tests, while Sects. 4 and 5 introduce the discussion and conclusion sections, respectively.

## Methods

The water-based air purifier is the second version of the semi-outdoor filterless air purifier, which uses a high-pressure 120 W air blower which produces 11.4 m^3^/h of air output [18]. The basic concept of both the old and new machines is the same, except that the air blower has been replaced by a dual duct ventilation fan 50 Hz 220 V 130 W, capable of releasing air at 900 m^3^/h and with a static pressure of 380 Pa, for a total of 1800 m^3^/h. Smart functions such as air quality, temperature, humidity, and an LCD monitor have been added to this water-based air purifier.

### Machine design

**Fig. 1:**
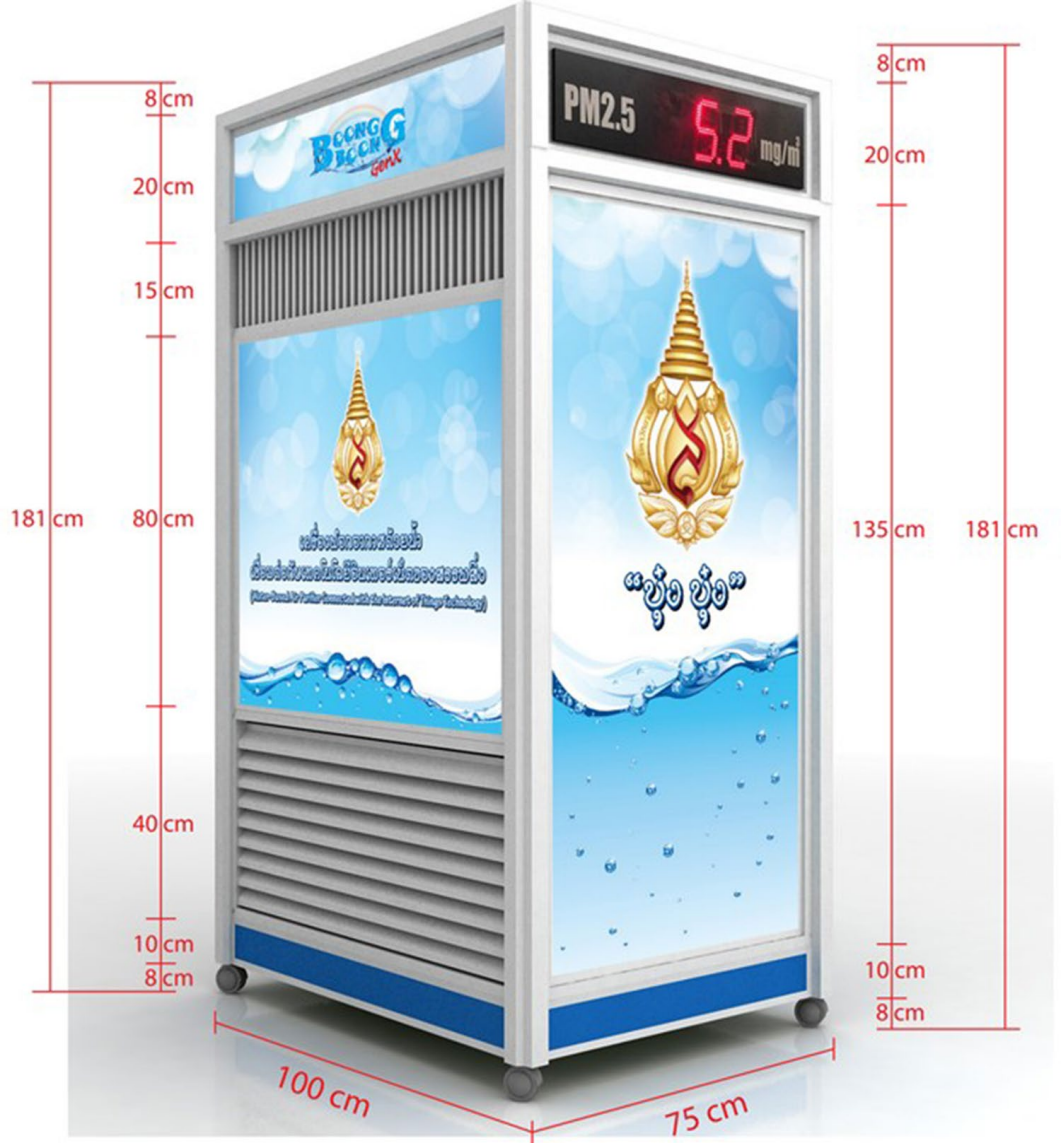
Outer dimensions of water-based air purifier. Bottom grills serve as an air inlet, whereas top grills represent as an air outlet

**Fig. 2:**
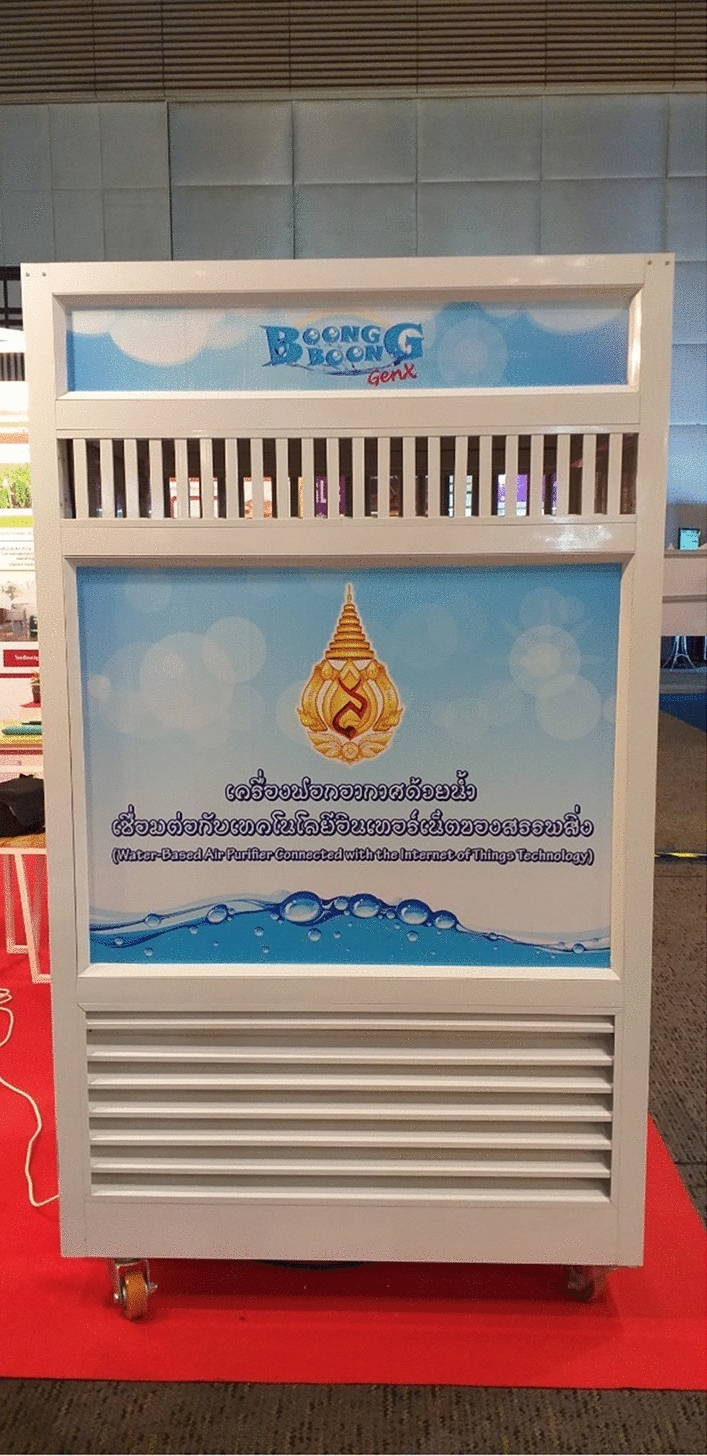
Water-based air purifier prototype

**Fig. 3:**
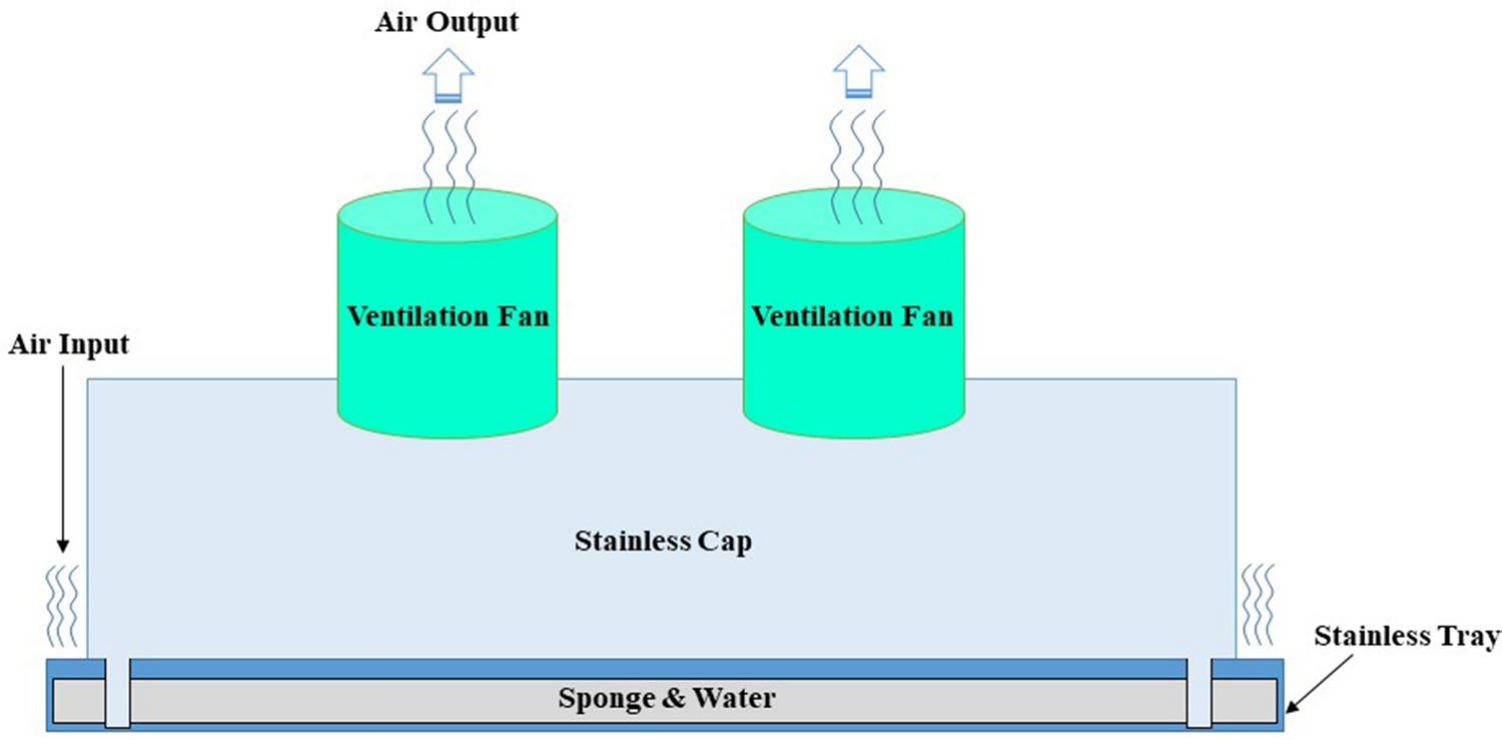
Working method of water-based air purifier prototype

**Fig. 4:**
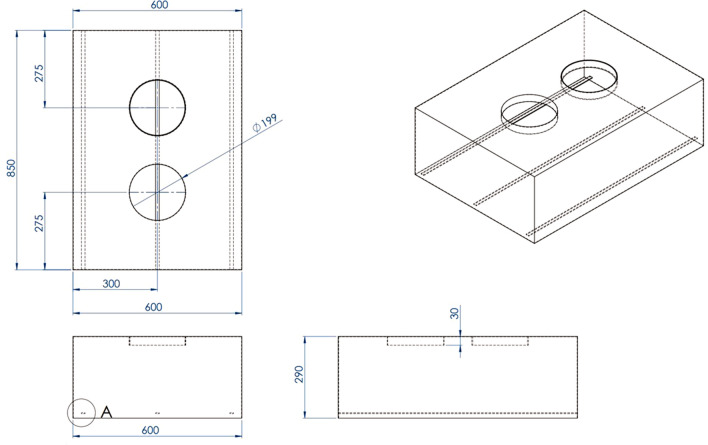
Dimensions of the stainless cap (millimeter)

**Fig. 5:**
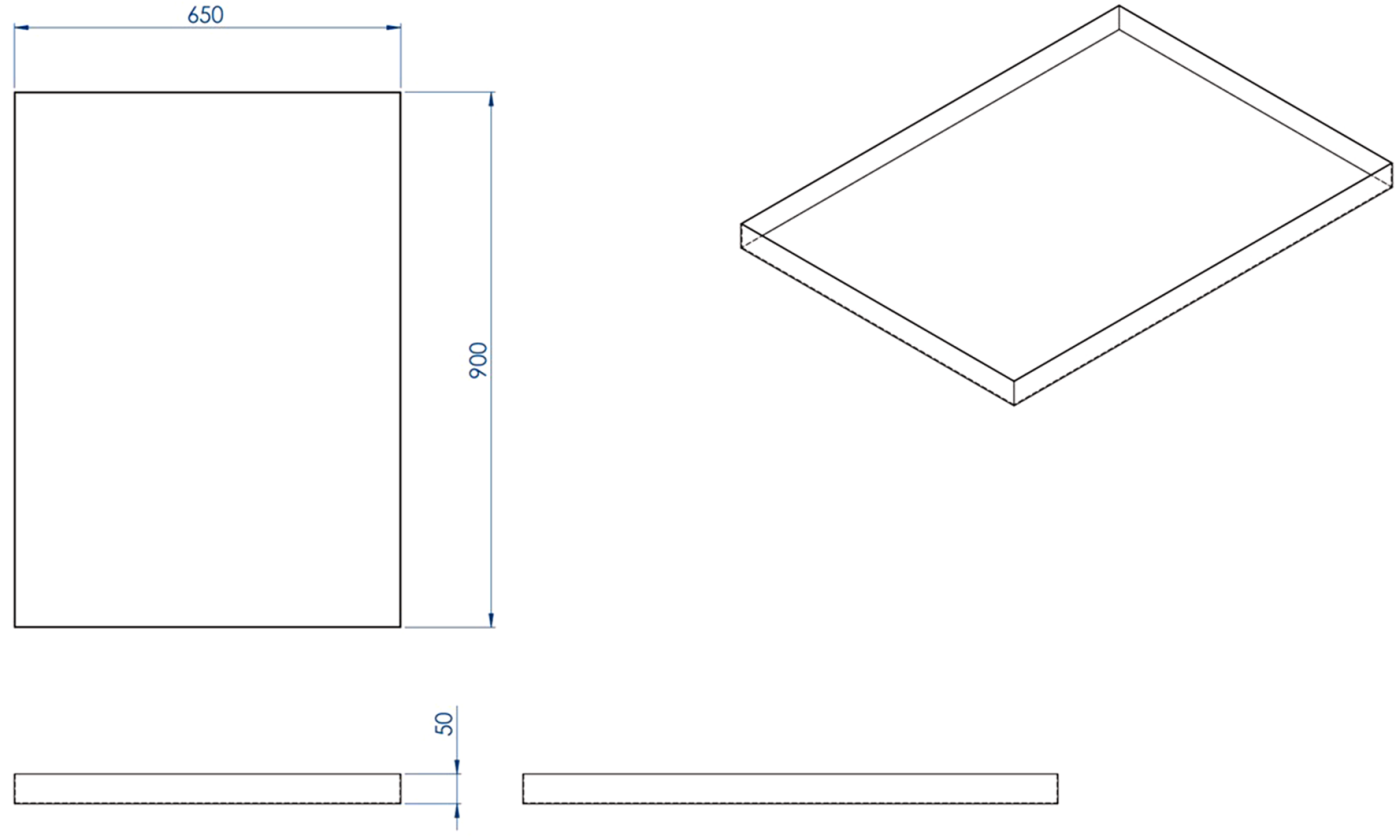
Dimensions of the water tray (millimeter)

Figure 1 depicts the outer dimensions of the water-based air purifier, which was primarily made of aluminum composite. As a result, it is light in weight and easy to transport. The structural components of the machine’s air purifying system is mainly made of 304 stainless steel sheets, with a thickness of 1.2 mm. After entering the gap between a water tray and a cap, dirty air is passed through a 0.5-mm capturing polyurethane sponge measuring 2.54 cm in thickness that is soaked with 5 Litres of water. The air flow is then directed to the inner side of the stainless cap, the dual ventilation fan, and finally back out into the surrounding environment. The prototype of the novel water-based air purifier is shown in Fig. 2. Figure 3 depicts how the water-based air purifying system works. Figures 4 and 5 illustrate the stainless cap and water tray drawings, respectively.

### Data collection and analysis

All tests were carried out at two locations: the first, a closed laboratory room measuring 11.7 × 16.2 × 3 (W × L × H) m^3^, and the second, a semi-outdoor space within a building, measuring 9 × 12 × 3 (W × L × H) m^3^. The Portable Particle Counter Model 9310 TSI AeroTrak^®^, which can calculate particle sizes of PM0.3, 0.5, 1.0, 3.0, 5.0, and 10 μm, was used to measure the amount of air pollution at 0, 5, 10, 15, and 20 min at the same locations, including the air inlet, air outlet, 2 m, and 4 m distance from one of the air inlets. Throughout the test, the water-based air cleaner was running continuously. Meanwhile, the Q-Trak Indoor Air Quality Monitor 7575 was used to measure CO_2_ (PPM) and relative humidity (RH). The results were expressed as a percentage difference in PM concentration versus haze particles at 0 min, when pollution was declared to be 100%. The average air speed was measured at four different locations in the vicinity of an air outlet using the Q-Trak Monitor, and the data was then averaged along with CO_2_ and RH. After 3 h of continuous machine work, the volume of water within the stainless tray was measured again.

## Results

In Table 1, as part of the PM mass concentration at 568.62 m^3^-indoor condition, PM from all distant locations, except PM0.3 at 4 m away from an air inlet, decreased after 20 min when compared to 0 min. PM ranging from 0.5 to 5 µm maximum decreased at 15 min, at the air inlet, which also remained 13.669 to 67.904%, while PM0.3 (91.839%) and PM10 (0%) levels were lowest at 10 min and 20 min at the air outlet, respectively. Large particles were cleaned better by the water-based air purifier than small particles. Overall, the best performance of the water-based air purifier was demonstrated at the 15-min air inlet measurement. The CO_2_ level was also at its lowest (92.789%) at this condition. The relative humidity ranged from 90.827 to 100.276% when compared to 0 min.

**Table 1:** The percentage differences in PM concentration, CO_2_, and relative humidity of indoor air when compared to 0 min

Location	Time (min)	PM mass concentration (%)						CO_2_ (%)	RH (%)
		PM0.3	PM0.5	PM1	PM3	PM5	PM10		
Air inlet	0	100	100	100	100	100	100	100	100
	5	98.558	82.164	62.665	41.068	57.554	240	97.154	93.540
	10	97.222	71.250	44.546	31.553	46.763	60	96.774	90.827
	15	97.079	67.904	39.099	13.856	13.669	40	92.789	91.990
	20	99.456	70.863	39.680	13.856	15.108	40	95.066	91.860
Air outlet	0	100	100	100	100	100	100	100	100
	5	95.548	79.236	65.819	52.155	64.706	200	97.697	93.309
	10	91.839	69.903	56.209	39.224	56.863	300	97.697	94.282
	15	93.503	69.678	51.401	25	25.490	50	95.010	93.309
	20	95.436	68.856	47.717	34.914	29.412	0	93.090	92.944
2 m from air inlet	0	100	100	100	100	100	100	100	100
	5	97.410	83.678	66.245	46.993	60	87.5	98.656	99.180
	10	93.705	72.464	50.410	20.490	20	87.5	95.969	97.268
	15	98.471	75.690	47.211	32.071	38.333	12.5	94.434	98.634
	20	98.342	74.728	43.995	23.831	28.333	12.5	93.282	96.585
4 m from air inlet	0	100	100	100	100	100	100	100	100
	5	101.233	89.071	72.380	109.385	103.571	366.667	96.518	98.204
	10	95.563	78.218	54.870	37.540	44.048	66.667	95.358	100.276
	15	98.457	79.607	50.970	44.660	40.476	66.667	98.066	99.033
	20	103.372	82.953	51.790	44.984	50	33.333	93.617	98.619

In Table 2, the air cleaning efficiency appeared to be lower in a semi-outdoor environment measuring 324 m^3^ than in an indoor condition. Only the 20 min measurement at the air outlet showed a reduction to 80 percent of PM10 when compared to the 100 percent control. PM from 1 to 5 µm maximum decreased at 10 min of 4 m distance from an air inlet, which also remained 57.209 to 88.525%, while PM0.3 (96.206%) and PM0.5 (92.045%) levels were lowest at 5 min of air inlet. At 20 min, CO_2_ levels were minimally elevated for all conditions (101.191 to 107.207%), whereas RH levels were slightly decreased (91.089 to 95.658%).

**Table 2:** The percentage differences in PM concentration, CO_2_, and Relative humidity of semi-outdoor air when compared to 0 min

Location	Time	PM mass concentration (%)						CO_2_ (%)	RH (%)
		PM0.3	PM0.5	PM1	PM3	PM5	PM10		
Air inlet	0	100	100	100	100	100	100	100	100
	5	96.206	92.045	92.070	66.071	91.346	125	103.820	95.332
	10	101.955	100.650	104.618	94.841	117.308	175	105.393	93.918
	15	99.417	92.248	92.712	74.802	113.462	325	102.023	89.816
	20	98.951	93.948	95.280	68.452	84.615	150	104.045	91.089
Air outlet	0	100	100	100	100	100	100	100	100
	5	100.575	103.299	97.931	91.688	112.941	140	101.099	96.567
	10	102.026	104.834	103.062	65.974	92.941	140	101.978	96.280
	15	100.678	94.882	90.087	102.078	138.823	220	111.648	93.419
	20	100.056	95.933	89.432	108.312	148.235	80	101.539	92.704
2 m from air inlet	0	100	100	100	100	100	100	100	100
	5	104.829	105.851	102.337	83.437	92.174	800	133.333	99.270
	10	102.269	100.943	97.877	76.190	98.261	1200	109.910	98.248
	15	102.072	97.587	95.192	75.569	79.130	300	104.955	96.058
	20	101.855	98.974	97.337	114.700	156.521	1700	107.207	95.328
4 m from air inlet	0	100	100	100	100	100	100	100	100
	5	101.565	101.445	96.564	82.326	105.921	150	101.191	100.145
	10	101.457	97.417	88.525	57.209	63.158	150	97.619	96.527
	15	101.526	98.561	94.443	80.310	101.316	137.500	101.191	97.250
	20	99.0163	96.167	92.491	73.333	93.421	137.500	101.191	95.658

The average airflow rate as part of an air cleaning volume was 0.0247 m^3^/s, which can treat an air volume of 88.92 m^3^/h. This volume was 20 times less than the total airflow specification of a dual ventilation fan (1800 m^3^/h). The volume of water within the stainless tray decreased from 5 to 4.5 L after 3 h of continuous machine work. As a result, the water evaporation rate of this air purifier in an indoor environment was approximately 0.167 L/h (17.3 to 22.9 °C, 70.1 to 82.2%RH).

## Discussion

According to the findings of this study, the use of the water-based air purifier in an indoor environment showed a significant difference when compared to a semi-outdoor environment. In an indoor environment, the target area of an air treatment system should be near both the air entry point as well as the air outflow point. This air purifier is suitable for gradual PM reduction, ranging from 0.5 to 10 µm, most effective after 15 min of machine operation. Therefore, it is appropriate for preventing dust as well as COVID-19 airborne particles in large spaces. Even though PM0.3 was not significantly reduced and SARS-CoV-2 genes were found in aerosols at 0.25–0.5 µm, data from an introduction section claims that the water-based air purifier can trap COVID-19 at respiratory particle levels measuring 4.7 µm. The water-based air purifier can emit humidity, which has no effect on the overall RH within a 568.62 m^3^ closed room or a 324 m^3^ semi-outdoor condition, and it also has a minor effect on CO_2_ levels. Despite the fact that the author only filled water to the upper surface of the polyurethane sponge, measuring 2.54 cm in thickness, the airflow rate dropped to 1/20 of the maximum efficiency of the air purifying system. As a result, for the next experiment, water-resistant pressure should be calculated. Natural air is one of the most important factors influencing the airflow rate in a semi-outdoor environment, where the site is approximately half that of an indoor testing condition. The use of this air purifier is then available for areas at least 4 m away from the air cleaning site. Uncontrolled variables, such as wind speed, the amount of space that the air occupies, and air pressure, were also involved during experiments as part of a semi-outdoor condition, and the results were not satisfactory. The fan size used in these experiments may not have been appropriate. The calculation between the specification of a ventilation fan and the available space should be mentioned in the next experiment.

The water-based air cleaner requires only a 260 W-dual ventilation fan and a time/day water observation as part of its low-tech and maintenance costs. Water measurement using a microcontroller and an ultrasonic sensor can be combined with the water-based air purifier to promote an automation system [19]. In comparison to the wet scrubber, the wet scrubber can remove any air pollutants using liquid droplets. As a result, this method necessitates the use of at least two mechanical systems to power the air purification process including, air blowers and sprayer systems [20]. Therefore, the air purifier employing the wet scrubber technique requires the use of more specialized technicians than the water-based air purifier. More research should be conducted to compare the effectiveness of those two methods in terms of cost effectiveness, air cleaning rate, and usability. Any limitations of this study also include, we do not have a dust particle counter that can measure at the level of COVID-19 diameters, so we cannot conclude that the air purifier has an effect on COVID-19 directly, and we do not know the viability of viruses after they sink into water. Accurate measuring instruments, as well as a viral viability test, should be recommended for future research.

## Conclusions

In summary, the prototype’s design concept also includes ventilation fans, a stainless tray, a stainless cap, a polyurethane sponge, and water. The novel water-based air purifier effectively traps air particles ranging in size from PM0.5 to 10 µm. For the next experiment, researchers should compare the efficacy of the wet scrubber technique to that of the water-based methods in all aspects. Water level and rate of water evaporation should be calculated to maximize the efficiency of this air purifier prototype. Finally, the author hopes that this low-tech machine will help the world combat both the COVID-19 pandemic and the air pollution crisis.
